# GM-CSF-Dependent Inflammatory Pathways

**DOI:** 10.3389/fimmu.2019.02055

**Published:** 2019-09-04

**Authors:** John A. Hamilton

**Affiliations:** ^1^The University of Melbourne, Department of Medicine, Royal Melbourne Hospital, Parkville, VIC, Australia; ^2^Australian Institute for Musculoskeletal Science (AIMSS), The University of Melbourne and Western Health, St. Albans, VIC, Australia

**Keywords:** cell survival, polarization, inflammation, pain, IRF4, CCL17

## Abstract

Pre-clinical models and clinical trials demonstrate that targeting the action of the cytokine, granulocyte macrophage-colony stimulating factor (GM-CSF), can be efficacious in inflammation/autoimmunity reinforcing the importance of understanding how GM-CSF functions; a significant GM-CSF-responding cell in this context is likely to be the monocyte. This article summarizes critically the literature on the downstream cellular pathways regulating GM-CSF interaction with monocytes (and macrophages), highlighting some contentious issues, and conclusions surrounding this biology. It also suggests future directions which could be undertaken so as to more fully understand this aspect of GM-CSF biology. Given the focus of this collection of articles on monocytes, the following discussion in general will be limited to this population or to its more mature progeny, the macrophage, even though GM-CSF biology is broader than this.

## Introduction

The glycoprotein, granulocyte macrophage-colony stimulating factor (GM-CSF) or CSF2, was originally defined as a hemopoietic growth factor based upon its ability to form colonies *in vivo* of granulocytes, and macrophages from bone marrow precursor cells (1). However, subsequently, it has been viewed more as a cytokine acting via a specific receptor, expressed mainly on myeloid cell populations, such as monocytes/macrophages, neutrophils and eosinophils, to enhance their survival and/or to activate/differentiate them (2–5). While not having a significant effect on steady state myelopoiesis, in the lung GM-CSF signaling normally maintains surfactant homeostasis and its disruption causes pulmonary alveolar proteinosis (PAP) most likely due to compromised alveolar macrophage development (6, 7). This GM-CSF-driven development of lung alveolar macrophages is of fetal monocyte origin (8). Recently it has been proposed that GM-CSF is required for cholesterol clearance in alveolar macrophages with reduced cholesterol clearance being the primary macrophage defect driving PAP pathogenesis (9). There is evidence that GM-CSF also controls non-lymphoid tissue dendritic cell (DC) homeostasis (10).

Seeing that this Review resides within a collection of articles on monocytes its content will generally be focussed on this population and its tissue counterpart, the macrophage, even though GM-CSF biology is broader involving other responding cell types such as neutrophils and eosinophils.

## GM-CSF and Autoimmune/Inflammatory Disease

Based mainly in data using GM-CSF gene deficient mice or neutralizing monoclonal antibody (mAb) in models of autoimmunity and chronic inflammation, it is apparent that GM-CSF can be a key driver of tissue inflammation and its associated pain. Examples include arthritis, EAE, cardiovascular disease, and lung disease. The data summarizing these findings have been reviewed recently (11–14) although some of this data more pertinent to the main topic of this Review will be mentioned. It should also be noted that systemically administered GM-CSF can have beneficial effects in inflammatory disease (for example, colitis) and host defense (for example, as an adjuvant) although caution should be exercised in assessing the significance of such administration for the role of endogenous GM-CSF in inflamed tissue (5, 14).

Given the potentially wide range of GM-CSF biology involving multiple cellular sources and responding myeloid cell types (5), human conditions that involve both acquired and/or innate immunity could fall within the realm of GM-CSF influence. As a result of some of the basic biology from pre-clinical models and GM-CSF expression in the corresponding human condition, a number of clinical trials using neutralizing mAbs to target GM-CSF or its receptor in autoimmune/inflammatory diseases have been performed and are continuing. There have been beneficial effects on disease severity in rheumatoid arthritis and asthma trials but, for reasons yet to be elucidated, not in plaque psoriasis—the data from these trials have been reviewed recently (11, 13–15).

## GM-CSF Receptor and Signaling

The GM-CSF receptor (GM-CSFR) is a type I cytokine receptor comprising in a multimeric complex a binding (α) subunit and a signaling (β) subunit, the latter shared with interleukin 3 (IL-3) and interleukin-5 (IL-5) receptors. These pathways have been linked to key residues in the intracellular regions of GM-CSFR using mainly receptor mutants expressed in cell lines (16–18). Key downstream signaling pathways from GM-CSFR are often those involving JAK2/STAT5 and ERK (16, 17, 19–21) with ERK activity linked to GM-CSF enhancement of human monocyte survival *in vivo* (21). The GM-CSF-driven development of lung alveolar macrophages is dependent on the transcription factors, PU.1 (22) and PPARγ (23). The debated contribution of other transcription factors, namely interferon regulatory factor (IRF) 4 and IRF5, to GM-CSF-driven monocyte/macrophage polarization (24–26), is discussed below.

The various cellular responses (survival, proliferation, activation and/or differentiation) appear to be explained by dose-dependent and sequential activation by GM-CSF of specific signaling pathways downstream of the activated receptor (16, 27). For example, physiological picomolar concentrations of GM-CSF are able to promote Ser585 phosphorylation in the cytoplasmic domain of the GM-CSFR β subunit to regulate cell survival via phosphoinositide 3-kinase activity and in the absence of other biological responses which occur at higher GM-CSF concentrations (18, 28). A time- and dose-dependent licensing process in mouse and human monocytes by GM-CSF *in vivo* has been described that disables their inflammatory functions and promotes their conversion into suppressor cells (29): this two-step licensing requires activation of the AKT/mTOR/mTORC1 signaling cascade by GM-CSF followed by signaling through the IFN-γR/IRF-1 pathway. Consistent with these dose-dependent signaling responses, dose dependent effects of a neutralizing anti-GM-CSF mAb on monocyte-derived activation/polarization vs. cell number levels were found in an inflammation model—indications were that higher local GM-CSF concentrations were needed for the activation/polarization response (30). Monocytes/macrophages generated *in vivo* from mouse bone marrow precursors with different concentrations of GM-CSF differed in function with possible implications for GM-CSF-dependent pathology (31)—cells generated with a high concentration of GM-CSF were more potent in generating cytokines and chemokines. The links between the various signaling pathways listed and their dependence on GM-CSF concentration in monocytes/macrophages requires further analysis to assess their contribution to the various cellular responses mentioned above. Additional signal transduction findings, particularly linked with the role of GM-CSF in inflammation, are described below.

## Cellular Sources of GM-CSF and “Networks”

Both hemopoietic [e.g., T and B lymphocytes (12, 32–35) and innate lymphoid cells such as ILC3] (36–38) and non-hemopoietic cell types (e.g., fibroblast, endothelial, and epithelial populations) can produce GM-CSF although usually requiring an activating stimulus (5, 12, 14, 32, 37, 39–43). In accord with this requirement, in the steady state GM-CSF circulates at low levels and tends to be expressed basally in non-sterile tissues such as skin, lung and gut (44, 45). Even though in inflammation GM-CSF can serve as a communication conduit between tissue-invading lymphocytes and myeloid cells, there is some controversy as to which factors can induce GM-CSF production in T helper (Th) cells (12).

To help understand the chronicity of certain inflammatory/autoimmune responses, a “CSF network” hypothesis was originally proposed in which there is an interdependent co-regulation of proinflammatory cytokines, such as IL-1 and TNF, with GM-CSF as part of a positive feedback “loop” involving communication between monocytes/macrophages and neighboring cell populations, such as fibroblasts, endothelial cells etc. (3–5, 46); this concept has been expanded to include cytokines, such as IL-23 and IL-6, as components of an autocrine/paracrine “network” involving macrophages, DCs and Th cells (45, 47, 48). Recently, positive feedback “loops” have also been put forward involving GM-CSF in inflammatory-dilated cardiomyopathy and breast cancer metastasis (49, 50).

## GM-CSF and Monocyte/Macrophage Function

### Macrophage Polarization

Based only on increased expression of pro-inflammatory cytokines, GM-CSF-treated monocytes/macrophages have been termed “M1-like” (51). However, such cells have also been considered to have characteristics of both M1 and M2 cells, for example, as regards their cytokine expression (39, 52), and GM-CSF-activated mouse monocytes have been reported to alleviate experimental colitis (52). Partly on account of the modest overlap with classical M1 polarization and their dual M1/M2 characteristics, it has been recommended that the M1/M2 polarization terminology not be applied to GM-CSF-treated monocytes/macrophages (14, 25, 26, 53). Even though increased mRNA expression for TNF, IL-1β, and IL-6 is readily observed in GM-CSF-treated (primed) monocytes/macrophages *in vivo*, significant cytokine secretion usually requires another stimulus, such as lipopolysaccharide (26, 54, 55).

Endogenous mediators can contribute to the phenotypes of GM-CSF-treated monocytes/macrophages (25). As an example, GM-CSF-mediated macrophage polarization of human monocytes *in vivo* has been reported to be modulated by endogenous activin A (25, 56); it also has been proposed that the GM-CSF-induced PPARγ expression in human macrophages is primarily regulated in this way (57). Endogenous TGF-β has also been invoked to have a similar role in the development and homeostasis of mouse alveolar macrophages (58). Since most, if not all, mediators involved in the host inflammatory response to injury and/or infection are endeavoring to be beneficial by restoring homeostasis, it is important to explore such a role for GM-CSF in its action on monocytes/macrophages.

### Monocytes, Macrophages, and DCs

It is debated as to whether GM-CSF can give rise to monocyte-derived DCs (MoDCs) *in vivo* or not (10, 14, 30, 59–61) even though GM-CSF, often in combination with IL-4, is widely used *in vivo* to generate mouse and human DC populations from bone marrow precursors and blood monocytes, respectively (20, 62–64). Two major types of GM-CSF-dependent phagocytes, termed macrophages and inflammatory DCs, have been claimed to have arisen *in vivo* from mouse CD209^−^ and CD209^+^ monocyte subsets (65)—their relationships to the *in vivo* generated populations (see below) also need further analysis. Mouse CD103^+^ DCs (also called cDC1) from different lymphoid and non-lymphoid tissues have distinct functional activities and there has been disagreement about the contribution of GM-CSF to their development *in vivo* (10, 14, 66, 67) with perhaps varying levels of GM-CSF helping to explain the discrepancies between different studies (68). Obviously, more work needs to be done to understand the role of GM-CSF in cDC development in the steady state and during inflammation. It has been proposed that the effector functions of GM-CSF-expanded myeloid cells *in vivo* are guided by their tissue microenvironment (69).

Mouse populations generated by GM-CSF from bone marrow precursors are heterogeneous with cells having both DC and macrophage features being observed—such features include surface markers, morphology, motility, antigen presentation, T cell activation, cytokine production, and gene expression profiles (51, 70–73); in fact their nomenclature is debated as to whether they should be termed DCs or macrophages (25, 73–75). As an advance on the use and interpretation of the data from such cultures, cell sorting strategies have isolated populations from them with macrophage and DC properties (73). Again the GM-CSF concentrations employed likely contribute to the phenotypes of the resulting populations (31). The *in vivo* relationships of the *in vivo* generated populations from mouse bone marrow and human monocyte cultures are not fully defined.

### Inflammation/Autoimmunity

**Figure 1:**
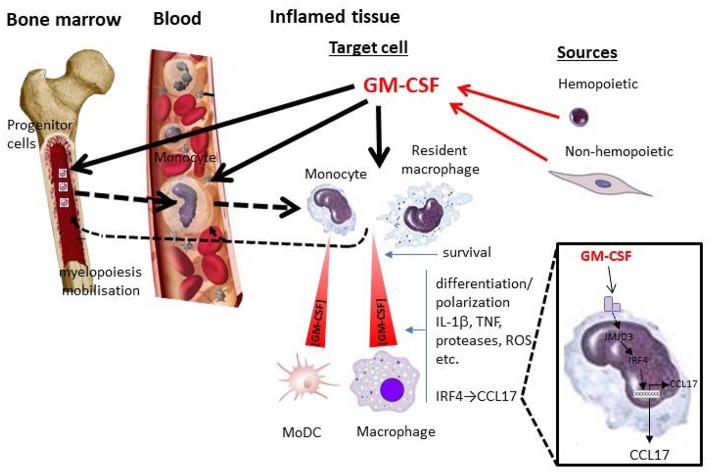
GM-CSF and monocytes/macrophages in inflammation. Depicted are some potential local and systemic actions of GM-CSF on monocyte/macrophage populations during an inflammatory reaction. Whether particular actions operate are currently debated and are likely to depend on the nature of the inflammatory reaction and the levels of GM-CSF attained from hemopoietic (e.g., lymphocyte) and non-hemopoietic (e.g., fibroblast) cell populations. Locally GM-CSF can act in a concentration—dependent manner on target cells (resident macrophages and/or blood-derived monocytes) to promote their survival and/or polarization/differentiation; the latter cell target can give rise to MoDCs. Their polarization/differentiation can be characterized by the production of proinflammatory mediators such as cytokines (e.g., IL-1β, TNF), proteases, reactive oxygen species (ROS), etc. One interesting pathway (zoomed), which seems to be important for GM-CSF-dependent inflammation and associated pain, leads to CCL17 production via JMJD3 and IRF4. GM-CSF can also act systemically in the blood and/or bone marrow, either directly or indirectly (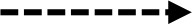) via its cellular targets in the tissue, leading to migration/mobilization of monocytes or their precursors and/or monocyte development from these precursors (myelopoiesis) (
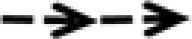
). MoDC, monocyte-derived DC.

In chronic inflammation and autoimmunity myeloid populations, for example, monocyte/macrophages and neutrophils, the cell populations which are potentially responsive to GM-CSF, are likely candidates to be regulating tissue damage and inflammation, being capable of releasing mediators, such as cytokines, chemokines, proteases and reactive oxygen species, as part of this response (5, 12, 26, 76, 77) (Figure 1). Of likely relevance to its function in inflammation/autoimmunity, GM-CSF upregulates class II MHC (21, 78, 79) and CD1 expression (80, 81) in human monocytes. However, it is worth noting that it cannot be assumed that monocytes/macrophages are the only myeloid cell types via which GM-CSF functions to regulate autoimmunity/inflammation (5). Amongst members of the macrophage lineage, GM-CSF initiates cardiac disease in resident mouse tissue macrophages (40) while in contrast only CCR2^+^ Ly6C^+^ monocytes require GM-CSF to lead to a pathogenic signature for EAE progression characterized by the induction of genes linked to inflammasome function, phagocytosis and chemotaxis, i.e., they become pathogenic DCs (76). Interestingly, it was reported that intrinsic GM-CSFR signaling by mouse monocytes and their precursors is not a prerequisite for the differentiation of monocytes into inflammatory monocyte-derived DCs *in vivo* during acute injuries (10). Nevertheless, moDCs do become more abundant in mice in which levels of GM-CSF are increased indicating again that GM-CSF can still be a critical factor influencing moDC differentiation, particularly under conditions where GM-CSF levels are elevated (61). GM-CSF-responsive CCR2^+^ moDCs and not Csf2rb^−/−^ moDCs are critical for Th17 induction and EAE progression (60). In addition to being able to preferentially control putative moDC numbers in antigen-induced mouse peritonitis, GM-CSF could also regulate macrophage numbers in the inflamed peritoneal cavity (30, 82, 83). Whether this regulation of monocyte-derived populations was due to effects of GM-CSF on cell trafficking in or out of a lesion and/or cell survival is unknown (30, 83) although effects on the latter parameter in other inflammatory/autoimmune models have been discounted (40, 60). Interestingly, in this context it has been suggested that GM-CSF controls mouse DC survival in non-lymphoid tissues as the mechanism for their homeostasis (10). There is also evidence that during an inflammatory response GM-CSF may act systemically to promote hemopoietic cell mobilization and development (40, 84–87).

### GM-CSF vs. M-CSF (CSF-1)

The gene expression profiles of human monocytes differentiated for 7 days in GM-CSF or M-CSF (CSF-1) differ substantially (25) and display distinct bioenergetic profiles (88). Since monocytes/macrophages are in general likely to be exposed to CSF-1 in the steady state, it has been proposed that pro-inflammatory stimuli, such as GM-CSF and interferon γ, lead to a cellular state of “CSF-1 resistance” or compromised CSF-1 signaling (5). CSF-1 could also be another endogenous mediator contributing to the phenotype of GM-CSF-treated human monocytes (89). Human monocytes differentiated in CSF-1 are widely used as a model for steady state tissue macrophages. In contrast to this widely used practice of employing CSF-1 as the differentiation stimulus, human monocytes treated *in vivo* with GM-CSF for 3 days have been used as a starting population of “macrophages” to analyse the transcriptional regulator networks upon cellular activation by a diverse range of stimuli (75, 90), stressing the need for researchers in the macrophage field to be conscious of the terminology used in any particular article.

### GM-CSF and Interferon Regulatory Factors (IRFs)

Based on a number of reports (91–94), the hemopoietic-specific transcription factor, IRF4 (95), appears to be a key signaling molecule regulating the adoption of DC-like properties in GM-CSF-treated precursors such as monocytes. Ly6C^hi^ Trem4^neg^ mouse monocytes can differentiate into Zbtb46^+^ MoDCs in response to GM-CSF and IL-4 in an IRF4 dependent manner (96). Also, GM-CSF-IRF4 signaling upregulates MHC Class II expression in mouse macropahges (97). However, IRF5 rather than IRF4, has been reported to be important for GM-CSF-mediated macrophage polarization (24) although there is disagreement with this conclusion in that IRF4 is considered to be more important based on the divergent data for the relative enhanced expression of the two IRFs by GM-CSF in human monocytes (25, 26). There is no obvious reason for this divergence although subtle differences in culture conditions could perhaps play a role. In support of the importance of IRF4, there is recent evidence that IRF4, most likely acting in monocytes/macrophages, is important in controlling how GM-CSF promotes arthritis and associated pain, as well as inflammatory pain *per se* (26, 98). There is evidence in turn that enhanced JMJD3 histone demethylase activity is required for GM-CSF-induced IRF4 transcription to occur in monocytes/macrophages as well as for GM-CSF-induced inflammatory pain (26) (see below).

### GM-CSF/CCL17 Axis

We recently found that the chemokine, CCL17, is the most highly up-regulated gene in GM-CSF-treated human monocytes and, unlike TNF and IL-1β, is secreted at high levels by GM-CSF-treated monocytes and mouse macrophages (26). It was also found surprisingly that CCL17 mediated GM-CSF-driven inflammatory pain as well as GM-CSF-driven and GM-CSF-dependent arthritic pain and disease. These pro-inflammatory actions of GM-CSF via CCL17 in turn required IRF4 and JMJD3 activity (26) (Figure 1). This proposed pro-inflammatory effect of IRF4 in macrophages was also surprising as IRF4 is usually considered to have an anti-inflammatory role in such cells since it down-regulates their production of pro-inflammatory cytokines such as TNF and IL-1β (99–101). Thus, GM-CSF joins the list of cytokines, such as IL-4 and TSLP, which can up-regulate CCL17 expression in monocytes/macrophages. This new GM-CSF → CCL17 pathway appears to be active in rheumatoid arthritis patients since circulating CCL17 levels are dramatically reduced upon anti-GM-CSF receptor monoclonal antibody therapy (102).

More recent studies have indicated that the GM-CSF → CCL17 pathway can be linked with TNF activity (103) as well as regulating experimental osteoarthritic pain and optimal disease (98)—the latter model data have led to a clinical trial being initiated in osteoarthritis using a CCL17 antagonist (NCT03485365 ClinicalTrials.gov). Interestingly, CCL17 may not necessarily be functioning as a chemokine in its regulation of inflammatory pain and arthritic pain/disease (98, 103).

## Conclusions

It would appear from the above that GM-CSF-dependent inflammatory pathways in monocytes (and macrophages) are likely to be critical for the purported role of GM-CSF in inflammation, autoimmunity and host defense. In addition to attempting to summarize the relevant literature on this topic I have tried to highlight some of the contentious issues which are currently being debated. Such issues, which I have endeavored to represent diagrammatically (Figure 1), are: (i) when, how and at what concentrations GM-CSF controls cell number and/or activation/differentiation (polarization) *in vivo*, (ii) whether GM-CSF controls MoDC development *in vivo*, (iii) the nature of GM-CSF-induced cell polarization, (iv) whether IRF4- or IRF5- dependent pathways are more important for GM-CSF-dependent biology, (v) when and how endogenous GM-CSF can act systemically in addition to locally in tissues, and (vi) how relevant are the effects of systemically administered GM-CSF to the actions of endogenous GM-CSF.

In order to understand better the role of GM-CSF-dependent pathways, future studies in some of the following areas are likely to be informative: (i) additional clinical trials targeting GM-CSF action and that of other putative downstream mediators, such as CCL17, (ii) human monocyte/macrophage studies, (iii) cellular metabolic responses to GM-CSF, and (iv) the significance of GM-CSF → IRF4 signaling.

## Author Contributions

The author confirms being the sole contributor of this work and has approved it for publication.

### Conflict of Interest Statement

The author declares that the research was conducted in the absence of any commercial or financial relationships that could be construed as a potential conflict of interest.
